# Persistence of West Nile Virus in the Central Nervous System and Periphery of Mice

**DOI:** 10.1371/journal.pone.0010649

**Published:** 2010-05-14

**Authors:** Kim K. Appler, Ashley N. Brown, Barbara S. Stewart, Melissa J. Behr, Valerie L. Demarest, Susan J. Wong, Kristen A. Bernard

**Affiliations:** 1 Wadsworth Center, New York State Department of Health, Albany, New York, United States of America; 2 Department of Biomedical Sciences, School of Public Health, University at Albany, Albany, New York, United States of America; University of Minnesota, United States of America

## Abstract

Most acute infections with RNA viruses are transient and subsequently cleared from the host. Recent evidence, however, suggests that the RNA virus, West Nile virus (WNV), not only causes acute disease, but can persist long term in humans and animal models. Our goal in this study was to develop a mouse model of WNV persistence. We inoculated immunocompetent mice subcutaneously (s.c.) with WNV and examined their tissues for infectious virus and WNV RNA for 16 months (mo) post-inoculation (p.i.). Infectious WNV persisted for 1 mo p.i. in all mice and for 4 mo p.i. in 12% of mice, and WNV RNA persisted for up to 6 mo p.i. in 12% of mice. The frequency of persistence was tissue dependent and was in the following order: skin, spinal cord, brain, lymphoid tissues, kidney, and heart. Viral persistence occurred in the face of a robust antibody response and in the presence of inflammation in the brain. Furthermore, persistence in the central nervous system (CNS) and encephalitis were observed even in mice with subclinical infections. Mice were treated at 1 mo p.i. with cyclophosphamide, and active viral replication resulted, suggesting that lymphocytes are functional during viral persistence. In summary, WNV persisted in the CNS and periphery of mice for up to 6 mo p.i. in mice with subclinical infections. These results have implications for WNV-infected humans. In particular, immunosuppressed patients, organ transplantation, and long term sequelae may be impacted by WNV persistence.

## Introduction

West Nile virus (WNV), a member of the Japanese encephalitis virus serogroup in the flavivirus genus of the family *Flaviviridae*, quickly spread across North America after its emergence in New York City in 1999 [1], [2]. In humans, WNV causes fever in approximately 20% of infections and neuroinvasive disease in <1% of infections [3], [4]. In the United States from 1999 through 2007, there were over 11,000 human cases of West Nile neuroinvasive disease and an estimate of over 1.5 million humans infected with WNV [5]. In addition, long term sequelae, including weakness, fatigue, and cognitive deficits, are observed in patients with both West Nile fever and West Nile neuroinvasive disease for up to 18 months after disease onset [6]–[18]. These sequelae are likely due to the initial damage caused by the virus, but viral persistence may also contribute to the extended recovery.

Members of the family *Flaviviridae* cause chronic infections, including hepatitis C virus and pestiviruses; however, members of the flavivirus genus are generally considered to cause acute infections. On the other hand, there is mounting evidence that these acute flavivirus infections can result in viral persistence. In convalescing humans, WNV RNA persists in the urine of patients for up to 6.7 years after disease onset [19]. In WNV-positive blood donors, WNV RNA is detected in blood for up to 104 days after index donation [20]. Other studies have examined the long term persistence of virus-specific immunoglobulin M (IgM), which is suggestive of viral persistence. Patients with West Nile disease and WNV-positive blood donors have persistent serum IgM for up to 11 to 16 months [20]–[23]. In addition, IgM persists in cerebrospinal fluid of patients with West Nile encephalitis for up to 5 months [24] and Japanese encephalitis for up to 6 months [25], [26], suggesting that flaviviruses can persist in the CNS of convalescing patients. In summary, these studies demonstrate that WNV persists in the periphery and possibly in the CNS of immunocompetent humans.

Flaviviruses and other arboviruses also persist in animal models (reviewed in [27]). WNV persists in experimentally inoculated animals, including macaques [28], hamsters [29], and house sparrows [30], for up to two to six months after inoculation. The goal of this study was to examine the persistence of WNV in immunocompetent mice with subclinical and clinical WNV infections. WNV persisted in the CNS and periphery of C57BL/6 (B6) mice as infectious virus for up to 4 mo p.i. and as RNA for up to 6 mo p.i.. This persistence occurred in mice with and without disease during the acute infection; therefore, West Nile disease was not required for viral persistence in the brain or spinal cord of mice. Viral persistence occurred in the face of a robust humoral response with WNV-specific antibodies persisting for at least 16 months. In addition, histologic lesions were observed in the brains of mice for up to 4 mo p.i., correlating with the presence of WNV RNA. Finally, transient immunosuppression with cyclophosphamide resulted in WNV recrudescence, suggesting that during viral persistence, the host's immune response prevents viral replication.

## Results

### WNV persists in mice

We previously showed that B6 mice are partially resistant to West Nile disease with approximately 30% morbidity and 20% mortality, and this resistance is not due to lack of neuroinvasion since WNV invades the CNS of all mice by 3 days p.i. [31]. Furthermore, infectious WNV was found in the CNS and skin of B6 mice for at least 14 days p.i. with mean viral titers of 10^3.9^ and 10^4.4^ PFU/g in the cerebral cortex and skin, respectively (Brown et al., unpublished data). These high viral loads late in infection led us to hypothesize that WNV persists in mice even without the development of disease. Thus, we conducted a study to test this hypothesis and to determine in which tissues and for how long WNV persists.

We examined persistence of WNV as infectious virus and RNA for 16 mo p.i., using our B6 mouse model. For this study, 82 mice were inoculated s.c. with WNV and assessed for clinical disease. Similar to previous results, the B6 mice exhibited 29% morbidity and 22% mortality. Mice were sacrificed at 1, 2, 3, 4, 6, 9 and 16 mo p.i., and we harvested tissues that are known targets for WNV [31], including skin, brain, spinal cord, lymph nodes, spleen, kidney, and heart. We used several methods to improve the sensitivity of our virus isolation (Figure S1). First, we used a co-culturing technique, which cultured the primary cells from the mouse tissues onto the highly susceptible Vero cell line. This technique amplified any virus that was produced from infected cells in the tissues. In addition, we “blind” passed the cultures two more times to further amplify any infectious virus. Finally, we tested all cell culture supernatants from the “third pass” even if no cytopathic effect (CPE) was observed, which allowed us to detect any non-cytopathic or slow growing virus. In two instances, WNV RNA was detected in the cell culture supernatant, and cytopathic WNV was isolated on a fourth pass (Table 1).

**Table 1:** Infectious virus and WNV RNA were isolated from at least one tissue in all mice at 1 mo p.i..

		Infectious Virus (WNV RNA) in tissue							
Mouse ID	Inoculum	Skin–inoculation site^1^	Skin–distal sites^2^	Spinal Cord	Brain	Lymph nodes^3^	Spleen	Kidney	Heart
7	Mock	− (−)	− (−)	− (−)	− (−)	− (−)	− (−)	− (−)	− (−)
57	WNV	+ (+)	− (+)	+ (+)	− (+)	− (+)	− (+)	− (+)	− (−)
58	WNV	+ (+)	− (−)	− (+)	− (+)	− (+)	− (+)	− (−)	− (−)
59	WNV	+ (+)	− (+)	− (+)	− (+)	− (+)	− (+)	− (+)	− (−)
60	WNV	+ (+)	+ (+)	− (+)	− (+)	− (−)	− (+)	− (−)	− (−)
63	WNV	− (+)	− (−)	− (+)	+ (+)	− (+)	− (+)	− (−)	− (−)
65	WNV	+ (+)	+^4^ (−)	− (+)	− (+)	− (+)	− (+)	− (−)	− (−)
67	WNV	+ (+)	− (−)	+ (+)	− (+)	− (−)	− (+)	− (−)	− (−)
90	WNV	+ (+)	− (+)	− (+)	− (+)	+ (+)	+^4^ (+)	+ (+)	+ (−)

− negative, + positive.

1 Skin at the inoculation site consisted of the left rear footpad.

2 Distal skin sites consisted of right rear footpad and both front footpads.

3 Lymph nodes consisted of both popliteal and both inguinal lymph nodes.

4 WNV was isolated on fourth pass.

Adult, female B6 mice were inoculated s.c. in the left rear footpad with diluent alone (mock) or with 10^3^ PFU of WNV. Mice were sacrificed at 1 mo p.i., and WNV infection was confirmed in all WNV-inoculated mice by seroconversion. Eight tissues per mouse were harvested for virus isolation and RT-PCR for WNV. Virus isolation was performed on eight tissues per mouse by co-culturing the homogenized tissues on Vero cell monolayers. All samples were passed at least two more times onto fresh monolayers, and tissue culture supernatants from the third passage were tested for the presence of WNV by RT-PCR. Mouse #90 was sick during acute phase of disease (7 to 14 days p.i.). All other mice did not show any clinical disease.

Using these sensitive techniques, we were able to isolate infectious WNV from at least one tissue in all mice at 1 mo p.i. (Table 1). Infectious WNV was most frequently isolated from the skin at the inoculation site with 88% of eight mice positive. WNV was isolated from all other tissues in at least one mouse (12 to 25%). After 1 mo p.i., infectious WNV was isolated in only one mouse, which was sacrificed at 4 mo p.i. (Table 2). Virus was isolated from three tissues of this mouse, the spinal cord, brain and spleen. This mouse had not been sick during the acute phase of disease (7 to 14 days p.i.), but it did show abnormal behavior for several weeks prior to sacrifice, including repetitive motions and over-grooming. It is unknown whether these behavioral abnormalities were associated with persistence of WNV; however, meningitis was observed on histopathology, suggesting that histologic lesions may contribute to neurologic sequelae.

**Table 2:** Summary of results for virus isolation, WNV RNA, and histopathology from mice sacrificed at various times p.i..

	Time post-inoculation (No. positive/No. tested)						
	1 mo	2 mo	3 mo	4 mo	6 mo	9 mo	16 mo
**Infectious WNV–any tissue** 1	8/8	0/8	0/9	1/8	0/8	0/8	0/15
**WNV RNA–any tissue** 1	8/8	8/8	8/9	3/8	1/8	0/8	0/15
**WNV RNA–skin**	8/8	7/8	5/9	1/8	0/8	0/8	0/15
**WNV RNA–spinal cord**	8/8	8/8	5/9	2/8	1/8	0/8	0/15
**WNV RNA–brain**	8/8	1/8	5/9	0/8	0/8	0/8	0/15
**Histologic lesions–brain**	7/8	5/8	ND	2/8	0/8	0/8	ND

ND = not done.

1 The number of mice with positive results from any of the eight tissues.

Adult, female B6 mice were inoculated s.c. in the left rear footpad with diluent alone (mock) or with 10^3^ PFU of WNV. Mice were sacrificed at the indicated times p.i., and each time point included one mock-inoculated mouse. WNV infection was confirmed in all WNV-inoculated mice by seroconversion at 1 mo p.i.. Eight tissues (brain, spinal cord, skin–inoculation site, skin–distal sites, spleen, lymph nodes, kidney, and heart) per mouse were harvested for virus isolation and RT-PCR for WNV. Virus isolation was performed by co-culturing the homogenized tissues on Vero cell monolayers. All samples were passed at least two more times onto fresh monolayers, and tissue culture supernatants from the third passage were tested for the presence of WNV by RT-PCR. Brains were examined for histopathology. All tissues from mock-inoculated mice were negative for infectious virus, WNV RNA, and histologic lesions.

**Figure 1:**
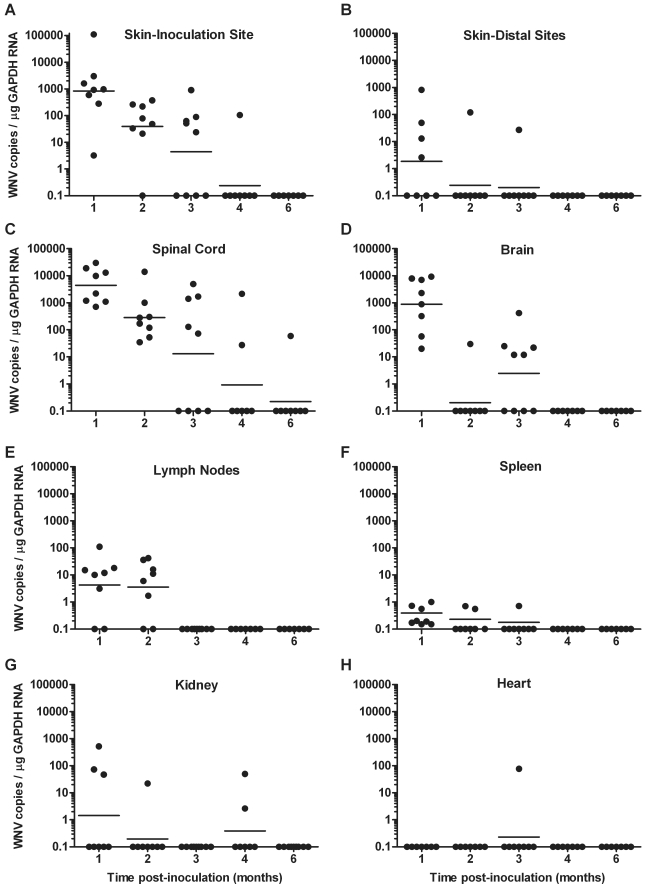
Persistence and levels of WNV RNA varies for different tissues. Adult, female B6 mice were inoculated s.c. with diluent alone (mock) or 10^3^ PFU of WNV in the left rear footpad, and tissues were harvested from 8 to 9 WNV-inoculated mice and one mock-inoculated mouse at various times p.i.. Levels of WNV genome copies per µg of GAPDH RNA were determined by real-time RT-PCR in (**A**) skin-inoculation site (left rear footpad), (**B**) skin-distal sites (right rear footpad and both front footpads), (**C**) spinal cord, (**D**) brain, (**E**) lymph nodes (both popliteal and inguinal lymph nodes), (**F**) spleen, (**G**) kidney, and (**H**) heart. Axis titles are the same for A–H. Each data point represents an individual animal, and the horizontal solid line is the geometric mean. Data points on the x-axis are negative. Data for mock-inoculated mice were negative for WNV RNA at each time point and are not shown on the graphs. Similar results were obtained in three independent studies performed at 1 mo p.i. (n = 8 to 10) and two independent studies performed at 2 mo p.i. (n = 4 or 8).

WNV RNA was detected in all mice and in seven different tissues at 1 mo p.i. although the frequencies and levels of WNV RNA varied for the different tissues (Table 1 and Figure 1). All samples of the skin at the inoculation site, spinal cord, brain and spleen were positive for WNV RNA, and the other tissues ranged from no positive samples (heart) to 75% (6/8) positive (lymph nodes) at 1 mo p.i. (Table 1). The highest WNV RNA levels were found in the skin at the inoculation site, spinal cord, and brain with geometric means of approximately 1000, 5000, and 1000 WNV copies/µg GAPDH RNA, respectively, at 1 mo p.i. (Figure 1A, 1C, and 1D). In contrast, the spleen had very low levels of WNV RNA (mean of 3 WNV copies/µg GAPDH RNA, Figure 1F), which may explain the difficulty in isolating infectious virus from this tissue despite the high frequency of WNV RNA present (Table 1). At 2 mo p.i., WNV RNA was again detected in at least one tissue of all mice (Table 2); however, the frequencies of detection declined for skin from distal sites, brain, spleen, and kidney (Figure 1B, 1D, 1F and 1G), and WNV RNA levels were lower in the skin at the inoculation site and spinal cord compared to 1 mo p.i. (Figure 1A and 1C). At 3 mo p.i., 89% of nine mice were positive for WNV RNA in at least one tissue, and skin at the inoculation site, spinal cord, and brain were most frequently positive at 56% (5/9, Table 2 and Figure 1). At 4 mo p.i., only 38% of eight mice were positive for WNV RNA, which was detected in skin at the inoculation site, spinal cord and kidney. At 6 mo p.i., only one mouse was positive for WNV RNA, which was detected in the spinal cord, and no WNV RNA was detected after this time. This study has been repeated three times at 1 mo p.i. (n = 8 to 10) and two times at 2 mo p.i. (n = 4 to 8), and very similar results were obtained for both virus isolation and WNV RNA in the various tissues (data not shown).

We evaluated the correlation between WNV persistence and the disease status of the animal during the acute phase of disease (7 to 14 days p.i.). Out of 64 surviving mice, six mice were sick during this time, and we sacrificed one sick mouse at each time point except for 4 mo p.i. The major reason for setting up the experimental design in this manner was to evenly distribute sick animals in order not to bias the results, but this design also allowed us to examine any effect of clinical disease. At 1 mo p.i., the sick mouse (ID #90) had five tissues positive for infectious WNV compared to only one to two positive tissues for the mice with subclinical infection (Table 1). At 2 mo p.i., the sick mouse had five tissues positive for WNV RNA compared to a range of only two to four positive tissues (mean +/− standard deviation = 3.2+/−0.89) for the mice with subclinical infection (data not shown). No differences were observed between mice with or without disease at later time points (data not shown). Although there was no significant correlation between WNV persistence and the disease status, there were more positive tissues in the sick animals at the earlier time points.

In summary, infectious WNV was isolated for at least 1 mo p.i. and at 4 mo p.i. in one of eight mice, and WNV RNA persisted for up to 6 mo p.i. Persistence of infectious virus occurred most frequently in the skin at the inoculation site. Persistence of WNV RNA occurred most frequently in the skin at the inoculation site, spinal cord and brain, followed by spleen, lymph nodes, kidney and heart in that order. The highest levels of WNV RNA were found in the skin at the inoculation site, spinal cord and brain, and WNV RNA persisted in these tissues for three, four and six months for the brain, skin at the inoculation site, and spinal cord, respectively. Overall these results demonstrate that persistence of WNV is tissue dependent, suggesting that the mechanisms and/or efficiency of viral clearance differ for the various tissues. Finally, the persistence of WNV was observed in mice with subclinical infection, demonstrating that disease is not required for WNV to persist in the CNS or peripheral tissues.

### WNV-specific antibody is long-lived in mice

Previous studies have shown that the persistence of antibodies to WNV varies in different animals–up to 14 months in humans [21], up to 15 months in horses [32], and up to 36 months in house sparrows [33]. Thus, we examined the longevity, quantity and quality of the antibody response in the mice from the viral persistent study described above, using microsphere immunoassays (MIA) and plaque reduction neutralization tests (PRNT).

**Figure 2:**
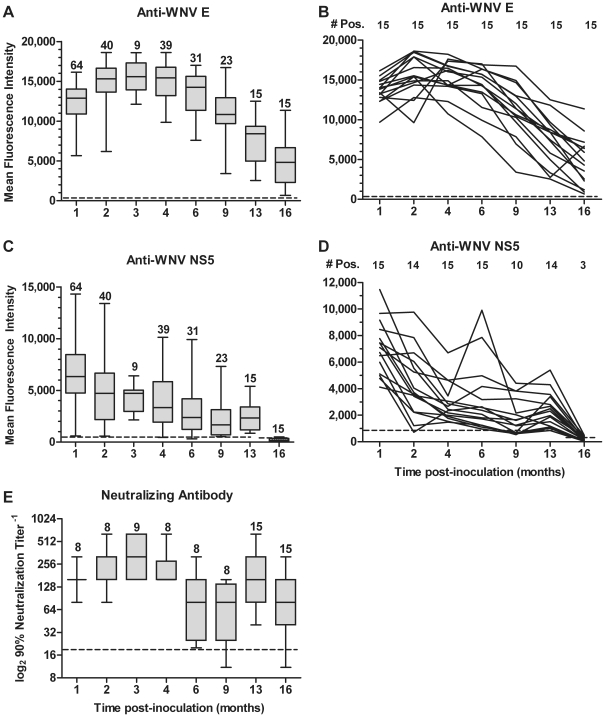
Mice maintain a robust antibody response during WNV persistence. Adult, female B6 mice were inoculated s.c. with diluent alone (mock) or 10^3^ PFU of WNV in the left rear footpad, and sera were harvested by serial tail bleeds or at time of sacrifice (Table S1 for details). Sera were tested for WNV-specific antibodies by (**A** and **B**) MIA for anti-WNV E, (**C** and **D**) MIA for anti-WNV NS5, and (**E**) PRNT with 90% endpoint titers. Axis titles are the same for A–D. The number of mice tested at each time point in A, C and E are placed above the whisker-box plots. In B and D, the same mice (n = 15) were bled at each time point, the results for an individual mouse over time is represented by a solid line, and the number of mice positive at each time point is listed at the top of the graphs. The horizontal, dotted lines correspond to the LOD, and data points below the dotted line are negative. Data for mock-inoculated mice were negative for WNV-specific antibodies in all assays and are not shown on the graphs.

We used MIAs, which are quantitative over a very broad linear range [34], to measure antibody levels to two WNV antigens, the structural envelope protein (E) and the nonstructural protein 5 (NS5). Sera were tested from mice that were sacrificed and from samples obtained by serial bleeding (9 to 64 mice at each time point, Table S1). The antibody response to E peaked at 2 to 4 mo p.i., and all mice remained positive for E antibody for at least 16 mo p.i. (Figure 2A). The antibody response to NS5 was highest at the first time point tested, 1 mo p.i. (Figure 2C). The antibody levels were evaluated from 15 mice that were serially bled over time. For antibodies to E, all 15 mice were positive at each time point, and the response plateaued at 2 mo p.i. (average mean fluorescence intensity [MFI] = 15,500) to 6 mo p.i. (average MFI = 14,200) and slowly declined between 9 mo p.i. (average MFI = 11,100) and 16 mo p.i. (average MFI = 4,800) (Figure 2B). In contrast, the antibody response to NS5 was more variable over time. All 15 mice were positive for antibody to NS5 through 6 mo p.i., but only 67% were positive at 9 mo p.i. (Figure 2D). At 13 mo p.i., 93% of the 15 mice were positive for antibody to NS5, and the average MFIs increased from 1,900 at 9 mo p.i. to 2,400 at 13 mo p.i. (*P* = 0.03, paired t-test). These results suggest that the response to NS5 was stimulated between 9 and 13 mo p.i.. By 16 mo p.i., there was a decline in the levels of antibody to NS5 with only 20% of the 15 mice positive and an average MFI of 190.

In addition to MIAs, we evaluated the levels of neutralizing antibodies to assess the functional antibody response. PRNTs were performed on sera from 8 to 15 mice per time point, which were from different mice at each time point except for 13 and 16 mo p.i. when the same 15 mice were tested (Table S1). Consistent with the kinetics of antibody to E (Figure 2A), the PRNT_90_ titers were highest at 2 to 4 mo p.i. with means of 1∶290 to 1∶370 and maximum values of 1∶640 (Figure 2E). The titers were lower at 6 and 9 mo p.i. with means of 1∶110 and 1∶80, respectively. The titers rebounded at 13 mo p.i. (mean of 1∶190), as was observed with the levels of antibody to NS5 (Figure 2D), but unlike the anti-NS5 levels, the PRNT titers were not from the same mice at 9 and 13 mo p.i., and the rise in titer at 13 months may be an artifact of the population sampled. By 16 mo p.i., the PRNT_90_ titers decreased to 1∶130 with 87% of 15 mice positive. When sera were evaluated using PRNT_50_ as an endpoint, all WNV-inoculated mice were positive for neutralizing antibody at all time points (data not shown). In summary, WNV-specific antibody persisted in mice for at least 16 months as measured by MIA for antibody to E, and all mice had detectable neutralizing antibody at this time. On the other hand, levels of antibody to NS5 were more variable over the course of the study and declined to undetectable or low levels at the end of the study.

### Histopathologic changes persist in the brain

**Figure 3:**
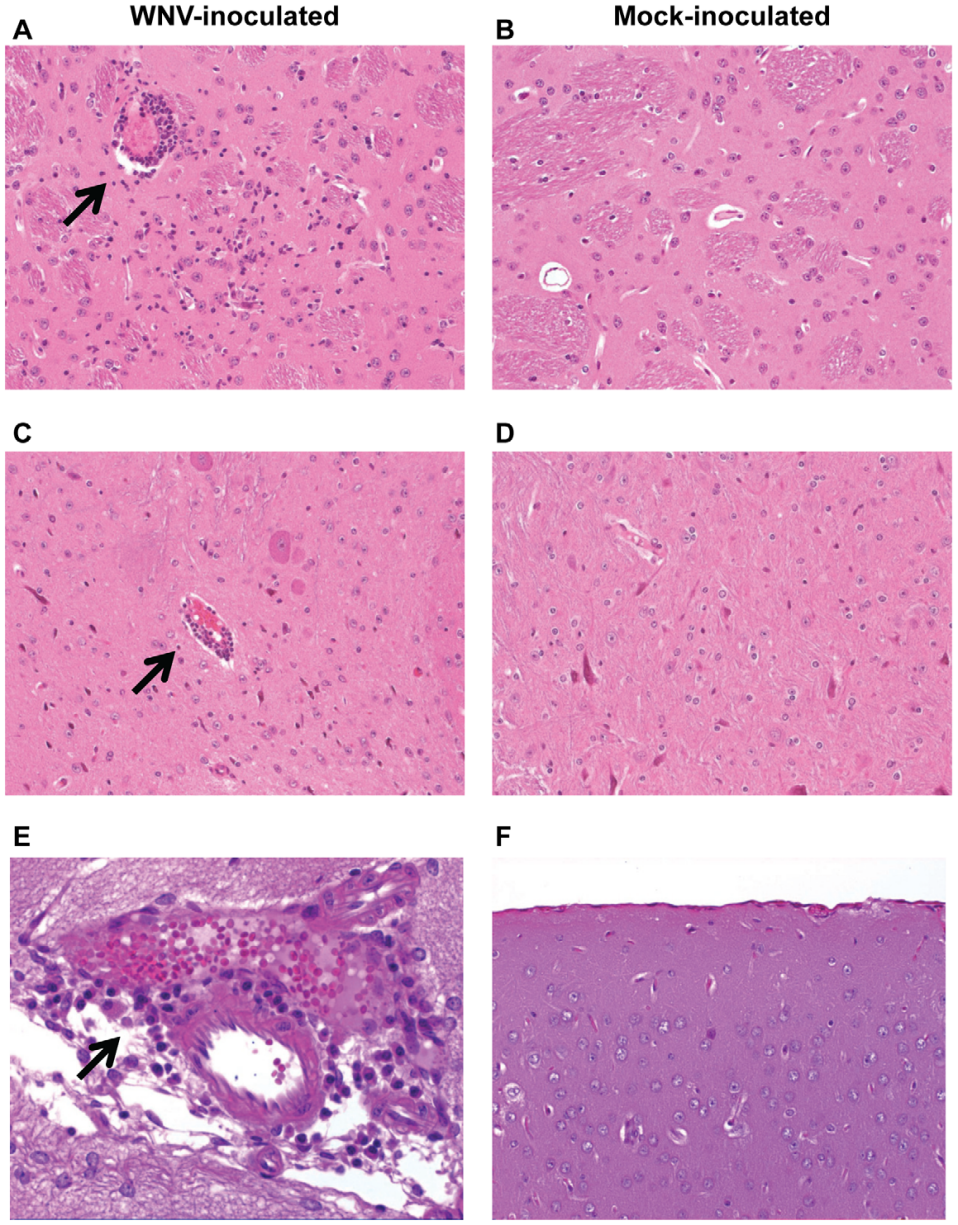
Histopathologic changes persist in the brains of mice during WNV persistence. Adult, female B6 mice were inoculated s.c. with diluent alone (mock) or 10^3^ PFU of WNV in the left rear footpad, and brains were harvested for histopathology from 8 WNV-inoculated mice and one mock-inoculated mouse at 1, 2, 4, 6 and 9 mo p.i.. Representative photomicrographs of brains are shown at (**A** and **B**) 1 mo p.i. in the thalamus, (**C** and **D**) 2 mo p.i. in the brainstem, and (**E** and **F**) 4 mo p.i. in the midbrain for WNV-inoculated mice (left panels) and mock-inoculated mice (right panels). Black arrows point to areas of inflammation. Sections were stained with hematoxylin and eosin, and photomicrographs are shown at a magnification of 200× (A, B, C, D, and F) and 400× (E).

WNV persisted in the brains of mice even without clinical disease; therefore, we questioned whether the mice lacked histologic lesions, which might explain the lack of disease and viral persistence. Thus, we performed histopathology on brains from mice sacrificed at 1, 2, 4, 6, and 9 mo p.i. (n = 8 per time point). Encephalitis and/or meningitis were observed in 88%, 62%, and 25% of the mice at 1, 2 and 4 mo p.i., respectively, and no lesions were observed at 6 or 9 mo p.i. (Table 2). In addition, the decline in histologic lesions in the brain at 4 to 6 mo p.i. correlated with the decline in WNV RNA (Table 2) and the decline in neutralizing antibody (Figure 2E). Representative examples of histologic lesions are shown in Figure 3. The encephalitis was characterized by foci of gliosis, neuronal loss, prominent vessels, and lymphoid perivascular cuffing (Figure 3A and 3C). The meningitis was characterized by focal lymphocytic and lymphoplasmacytic infiltrates in the meninges (Figure 3E). In summary, histologic lesions were observed in the brain for up to 4 mo p.i. in mice with and without previous clinical disease, suggesting that inflammation in the brain does not necessarily cause clinical disease. Furthermore, these results provide evidence for an immune response in the brains of mice in the face of viral persistence.

### Immunosuppression results in viral recrudescence

Since we observed inflammation in the brains of mice with persisting WNV, we hypothesized that generalized immunosuppression after the establishment of viral persistence would allow viral replication (i.e. viral recrudescence). Thus, we treated WNV-inoculated mice at 28 days p.i. with cyclophosphamide, which transiently suppresses actively dividing lymphocytes [35], or with vehicle alone [phosphate buffered saline (PBS)]. One group of treated mice was sacrificed to examine tissues for persistent WNV, and another group was monitored for clinical disease. Three mice per treatment (cyclophosphamide and PBS) were sacrificed twelve days after the last treatment, and six tissues (skin at the inoculation site, spinal cord, brain, lymph nodes, spleen, and kidney) per mouse were examined for WNV by virus isolation and RT-PCR. Infectious WNV was isolated from three of three mice (two to five tissues per mouse) treated with cyclophosphamide and from none of three mice treated with PBS (Table 3), which was statistically different (*P* = 0.05, Fisher's exact test). Overall 9 of 18 tissues were positive in the cyclophosphamide-treated group, which is significantly different than no positive tissues in the PBS-treated group (*P* = 0.0005, Fisher's exact test). Detection of WNV RNA was not different for the two treatment groups with 8 of 18 tissues (44%) positive for both groups (Table 3). No disease was observed in the remaining mice for at least 3 mo p.i. (data not shown), suggesting that the immunosuppression was too transient for disease to occur and/or the presence of WNV-specific antibody (Figure 2) prevented disease. Furthermore, these results support our findings that WNV persists as fully infectious virus at 1 mo p.i. (Table 1).

**Table 3:** Treatment of mice with cyclophosphamide resulted in recrudescence of WNV.

			Infectious Virus (WNV RNA) in tissue					
Mouse ID	Inoculum	Treatment on 28 and 33 days p.i.	Skin–inoculation site^1^	Spinal Cord	Brain	Lymph nodes^2^	Spleen	Kidney
2	Mock	Cyclophosphamide	− (−)	− (−)	− (−)	− (−)	− (−)	− (−)
9	WNV	Cyclophosphamide	+ (+)	+ (+)	− (−)	− (−)	− (−)	− (−)
10	WNV	Cyclophosphamide	− (+)	+ (+)	+ (+)	− (−)	− (−)	− (−)
12	WNV	Cyclophosphamide	+ (+)	+ (+)	+ (+)	+ (−)	− (−)	+ (−)
25	WNV	PBS	− (+)	− (+)	− (−)	− (+)	− (+)	− (−)
26	WNV	PBS	− (+)	− (+)	− (−)	− (+)	− (−)	− (−)
27	WNV	PBS	− (+)	− (−)	− (−)	− (−)	− (−)	− (−)

− negative, + positive.

1 Skin at the inoculation site consisted of the left rear footpad.

2 Lymph nodes consisted of both popliteal and both inguinal lymph nodes.

Adult, female B6 mice were inoculated s.c. in the left rear footpad with diluent alone (mock) or with 10^3^ PFU of WNV. Mice were treated with cyclophosphamide or PBS on days 28 and 33. Mice were sacrificed on day 45 p.i., and WNV infection was confirmed in all WNV-inoculated mice by seroconversion. Six tissues (skin–inoculation site, spinal cord, brain, lymph nodes, spleen, and kidney) per mouse were harvested for virus isolation and RT-PCR for WNV. Virus isolation was performed by co-culturing the homogenized tissues on Vero cell monolayers. All samples were passed two more times onto fresh monolayers, and tissue culture supernatants from the third passage were tested for the presence of WNV by RT-PCR. Mouse #12 and mouse #25 were sick during acute phase of disease (7 to 14 days p.i.). All other mice did not show any clinical disease.

## Discussion

In this study, we have demonstrated that WNV persists in the CNS and peripheral tissues of mice for up to 6 mo p.i.. Furthermore, this persistence was observed in the CNS of mice that did not exhibit disease during acute infection (i.e. subclinical infection), which is consistent with our previous results that neuroinvasion occurs in mice with subclinical infection [31]. The frequency and levels of WNV persistence was tissue dependent. WNV persisted most frequently as infectious virus in the skin and as RNA in the skin, spinal cord, and brain. WNV persistence was relatively frequent in lymphoid tissues, was uncommon in the kidney, and rare in the heart. In addition, WNV persistence occurred in the face of a strong antibody response and active inflammation, and upon transient immunosuppression, the virus recrudesced.

Persistence of WNV in the CNS was previously reported in immunocompetent animals, but our results differ in the duration of viral persistence and disease status of the animals. WNV persists as infectious virus in brains of hamsters for up to 53 days [36] and brains and spinal cords of macaques for up to 6 months [28]. Both of these studies examined animals that were convalescent, and in the macaque study, WNV was inoculated intracranially, which may have affected persistence of WNV in the CNS. In contrast, we observed infectious WNV for up to 4 months in the brain and spinal cord after a peripheral inoculation (compared to approximately 2 months in hamsters), and WNV persisted in the CNS of mice with subclinical infection. In house sparrows, WNV RNA persists in the brain for up to 30 days [30] whereas we observed WNV RNA in the brains and spinal cords of mice for up to 3 and 6 months, respectively. Furthermore, we observed histologic lesions in the brains of mice for up to 4 months, which to our knowledge is the only report of brain pathology past the acute phase of West Nile disease in any animal model. In summary, we observed pathologic changes and longer duration of WNV persistence in the CNS of mice after peripheral inoculation compared to hamsters [36] and house sparrows [30], and unlike the macaques [28] and hamsters [36], the mice had subclinical WNV infections.

Persistence of WNV in skin has been examined in only one other published report, which studied persistence in house sparrows. WNV RNA persisted in the skin of 10 out of 13 house sparrows for 30 days, but infectious virus was not detected in the skin using plaque assays [30]. In contrast, we observed WNV RNA and infectious virus at 30 days in 100% and 88% of mice, respectively. Our ability to detect infectious virus may be due to the use of the more sensitive co-culturing technique to isolate virus. Furthermore, we detected WNV RNA in the skin for up to 4 months p.i., three months longer than was observed in house sparrows. The longer duration may be due to greater initial viral infection or different cell targets in the skin of mice compared to house sparrows. Alternatively, the footpad is thick skin, which is richly innervated and has a high density of mechanoreceptors, and it may support greater viral persistence than thin skin on other areas of the body. In addition, our results demonstrate that WNV persistence is less frequent in skin distal from the inoculation site. It is possible that dissection of the footpad ensured that we obtained the inoculation site, which would be more difficult to identify on the breast skin of a house sparrow [30].

Persistence of WNV in other peripheral tissues has been documented in several animal species. Hamsters inoculated with NY99 strain of WNV have persistent viruria for up to 52 days in 60% of the animals [29]. Although we did not examine urine in our studies, it is unlikely that we would have observed frequent viruria since only 25% of mice were positive for WNV RNA in their kidneys through 60 days. In another study, Tesh et al. [37] used an attenuated, hamster-adapted strain of WNV and observed persistence of infectious virus in kidneys and urine for up to 247 days. Unlike our observations in mice, WNV infrequently persists in spleens of hamsters [29], [37]. In macaques, infectious WNV was observed in kidneys, spleens and lymph nodes for up to 161 days after immunosuppression with cyclophosphamide [28]. In wild bird species, WNV RNA is detected in kidneys and hearts more frequently at 30 days [30] and in kidneys at 6 weeks [38] than we observed in mice; however, WNV persistence in spleens was similar for mice and birds [30], [38]. In summary, the frequency, duration, and tissue location of WNV persistence are species dependent. This dependence is most likely due to differences in the host's immune response, the severity of disease, initial viral loads, tissue tropism, and cell targets.

Other factors may contribute to the differences observed in persistence between various animal models, including viral strain, viral dose, and route of inoculation. In our study and the house sparrow study [30], 10^3^ PFU of WNV was inoculated subcutaneously. In the hamster study [36], 10^4^ PFU of WNV was inoculated intraperitoneally. All three studies used strains of WNV belonging to the NY99 genotype; however, our virus was derived from an infectious clone and was likely more homogenous. In the macaque study [28], ten strains of WNV were inoculated intracranially or subcutaneously at 10^6^ to10^7^ suckling mouse lethal dose-50. These strains were isolated over 30 years ago and have varied passage history; therefore, they are likely very divergent from the NY99 genotype of WNV.

Persistence of flaviviruses also occurs in human patients recovering from neuroinvasive disease (reviewed in [27]). In a recent longitudinal study of convalescing humans, 20% of the patients had detectable WNV RNA in their urine for 1.6 to 6.7 years after disease onset [19]. The duration of WNV persistence is much longer than what has been observed in other animals or in our studies of mice, and the long duration is likely influenced by the initial disease severity of these patients, who were hospitalized with neuroinvasive disease. In our study, mice with neuroinvasive disease were euthanized during acute illness (7–14 days p.i.). Other evidence of persistence in humans is the long duration of IgM, which suggests that virus and/or viral antigen is persisting. Patients with confirmed West Nile encephalitis have persistent serum IgM against WNV for up to 16 months [23]; patients with West Nile encephalitis [24] and Japanese encephalitis [25], [26], a related flavivirus, have persistent virus-specific IgM in their cerebrospinal fluid for up to 5 to 6 months, suggesting that flaviviruses can persist in the CNS of convalescing patients.

WNV also persists in humans after mild febrile illness or subclinical infections. In a longitudinal study of WNV-positive blood donors, 3% of the donors had detectable WNV RNA in blood between 40 and 104 days after their index donation, using a very sensitive transcription-mediated amplification technique [20]. Prince et al. [22] followed up WNV-positive blood donors, and serum IgM against WNV persisted for up to 1 year, suggesting that virus and/or antigen is persisting in these donors. The population of WNV-positive blood donors is representative of the general population infected with WNV in nature (80% subclinical infections, 20% West Nile fever, and <1% neuroinvasive disease) [22], [39]. Thus, as occurred in our B6 mouse model, there is strong evidence that WNV persistence occurs in humans with subclinical infections.

We observed WNV persistence in our mouse model in the face of a robust antibody response and inflammation in the brain. Transient immunosuppression with cyclophosphamide resulted in viral recrudescence as evidenced by the isolation of infectious WNV, suggesting that without actively dividing lymphocytes, WNV can replicate. Similar results were observed upon cyclophosphamide treatment of macaques inoculated with WNV [28] and mice inoculated with Japanese encephalitis virus [40]. These results suggest that WNV is persisting as infectious virus for months after infection. In addition, the variability of the antibody levels to WNV NS5 in our mice suggests that persistent WNV replicates periodically, produces non-structural proteins, and stimulates the immune response. The presence of fully infectious virus during persistence has clinical implications for humans who are persistently infected with WNV and subsequently become immunosuppressed.

We propose that after the establishment of viral persistence, there is a smoldering viral infection that the immune response keeps in check, but clears very slowly. Final viral clearance likely depends on the cell type infected with longer persistence in cells with slow turnover or cells such as neurons that the body must protect from damage. Viral persistence is likely greater in tissues with less immune surveillance, such as the CNS, which is supported by the more frequent persistence in the CNS of our mice. Even within the nervous system, there may be differences in clearance. We observed longer persistence in the spinal cord than in the brain, suggesting different clearance mechanisms in these sites, such as was observed with Sindbis virus [41]. The initial disease status also likely affects WNV persistence due to differences in initial viral load and/or influences on the early immune response. We observed a trend toward greater WNV persistence in mice that survived clinical disease, and persistence of virus and IgM in humans appears longer in patients recovering from West Nile neuroinvasive disease [19], [23] than in blood donors with mild or inapparent disease [20] although direct comparisons have not been made.

The persistence of WNV has implications for human health. Transmission of WNV occurs with organ transplantation from acutely infected donors [42] and may possibly occur with persistently infected donor organs in combination with immunosuppression of the recipient. In addition, it is unknown if WNV persistence contributes to the long term sequelae observed in patients recovering from West Nile encephalitis and West Nile fever [6]–[18]. There have been over 11,000 human cases of West Nile neuroinvasive disease and estimates of over 300,000 cases of West Nile fever and over 1.2 million humans with asymptomatic infections of WNV in the United States through 2007 [5]. The potential impact on this population is great. Future studies are needed to develop a model for long term sequelae and to further our understanding of WNV persistence, including studies on mechanisms of persistence and the immune response during viral persistence.

## Materials and Methods

### Ethics statement

All studies were approved by the Institutional Animal Care and Use Committee of the Wadsworth Center under protocol #06-377 and followed criteria established by the National Institutes of Health.

### Cells and virus

African green monkey kidney cells (Vero; ATCC #CCL-81) and baby hamster kidney cells (BHK-21; ATCC #CCL-10) were maintained in supplemented medium [minimum essential medium (Gibco® Invitrogen, Carlsbad, CA) with 5% fetal bovine serum, 2 mM L-glutamine, 1.5 g/L sodium bicarbonate, 100 U/ml of penicillin, and 100 µg/ml of streptomycin]. Cells were incubated at 37°C, 5% CO_2_.WNV was produced from a full-length cDNA clone of a 2000 New York strain (NY99 genotype) by electroporation of BHK-21 cells with *in vitro* transcribed RNA as previously described [43]. Viral titers of stocks were determined by plaque assay on Vero cells.

### Mouse studies

Five-week-old, female B6 mice were purchased from Taconic (Germantown, NY), acclimatized for at least 1 week in the BSL-3 animal facility, and given food and water ad libitum. For virus inoculation, six- to seven-week-old B6 mice were inoculated s.c. in the left rear footpad as previously described [31] with diluent alone (mock) or 10^3^ PFU of WNV. The diluent for viral inocula was endotoxin-free PBS (tissue culture grade; Invitrogen) with 1% fetal bovine serum. In this B6 mouse model, the lethal dose-50% is greater than 10^5^ PFU (Bernard, unpublished data), and the infectious dose-50% is 1 PFU [44]. After inoculation, all mice were observed for clinical disease daily for the entire study. All mice were weighed daily for at least 14 days p.i., three times per week for the 3rd and 4th weeks p.i., and once per week for the remainder of the study. Clinical signs included ruffled fur, hunching, ataxia, and weakness. A mouse was considered to have clinical West Nile disease if at least one of the following criteria was met: 1) ≥10% weight loss; 2) clinical signs for at least two days. Mice that exhibited severe disease were euthanized. No clinical signs or weight loss were observed in mock-inoculated mice. At 1 mo p.i., all WNV-inoculated mice were seropositive for WNV.

### Viral persistence study

A study to assess viral persistence in mice was performed using a single cohort of B6 mice. Mice were inoculated with diluent alone (n = 8) or WNV (n = 82) and monitored as described above. One mock-inoculated mouse and eight to nine WNV-inoculated mice were sacrificed at 1, 2, 3, 4, 6, and 9 mo p.i., and tissues were harvested (see below). At all time points except 4 mo p.i., one mouse was sacrificed that was previously sick during the acute phase of disease (7 to 14 days p.i.), and the other seven to eight WNV-inoculated mice had subclinical infection (i.e. no clinical signs of disease). All mice were bled at 1 mo p.i., and infection was confirmed in all WNV-inoculated mice by seroconversion, using an ELISA for WNV as previously described [31]. Mice that were not sacrificed were serially bled for serologic assays at 2, 4, 6, 9, and 13 mo p.i.. At 16 mo p.i., the remaining 15 WNV-inoculated and 2 mock-inoculated mice were sacrificed, and tissues were harvested (see below). Table S1 summarizes the study design.

### Tissue harvesting and processing

The following tissues were harvested at each time point: serum, skin–inoculation site (left rear footpad), skin–distal site (right rear and front footpads pooled together), lymph nodes (both popliteal and both inguinal lymph nodes pooled together), spleen, heart, kidney, spinal cord, and brain. The brain was cut on the median plane; one-half was processed for virus isolation, and the other half was processed for histopathology (see below). Tissues were processed as previously described except that tissues were not frozen [31]. Briefly, tissues were harvested and weighed, and BA-1 diluent (M199, 1% bovine serum albumin, 0.05 M Tris pH 7.6, 0.35 g/L sodium bicarbonate, 100 U/ml penicillin, 100 µg/ml streptomycin, 1 µg/ml fungizone) was added to make a 20% homogenate for all CNS tissues and a 10% homogenate for all other tissues. For small tissues, a minimum of 250 µl of BA-1 was added.

### Virus isolation

On the same day as tissue harvesting, the homogenized tissues were immediately processed for virus isolation as follows (see flow chart in Figure S1). Tissue homogenate (100 µl) was inoculated onto Vero cells in a 6-well plate (“pass 1”). Cultures were incubated at 37°C, 5% CO_2_ for 4 to 5 days and were observed daily for CPE. All samples were passed at least one more time (“pass 2”) to ensure that any CPE was not due to toxicity caused by the sample. After the second pass, all CPE-negative samples were passed one more time (“pass 3”). The second and third passes were done by inoculating 500 µl cell culture supernatant onto Vero cells in 6-well plates. Real-time RT-PCR assays (see below) were performed on all CPE-positive samples to confirm the presence of WNV, and assays for both WNV E and NS5 genes were positive. In order to detect slow growing or non-cytopathic virus, real-time RT-PCR assays that targeted both WNV E and NS5 genes were performed on all samples that were negative for CPE on “pass 3”. The few “pass 3” samples that were CPE-negative and WNV RNA-positive were passed a fourth time on Vero cells.

### RNA extraction

On the same day as tissue harvesting, tissue homogenate (50 µl) was mixed with 350 µl of RLT lysis buffer (Qiagen, Valencia, CA) and placed at −80°C until RNA extraction was performed (Figure S1). RNA was extracted from tissues and cell culture supernatants, using RNeasy Mini Kit per the manufacture's protocol (Qiagen). Extreme care was taken to avoid contamination and false positive results. For example, RNA extractions of CPE-positive cultures were performed on different days than extractions of tissues or CPE-negative cultures. No contamination was observed for tissues from mock-inoculated animals or for cell culture supernatants that were inoculated with tissues from mock-inoculated mice. In addition, reagent controls were run with every RNA extraction, and no contamination was observed.

### Real-time RT-PCR

WNV RNA was quantified using real-time RT-PCR assays for WNV as previously described [45], [46] with the following details. Two different primer/probe sets for WNV were used in two separate reactions, using TaqMan® One-Step RT-PCR (Applied Biosystems, Inc., Foster City, CA). The TaqMan® probes contained a 5′ 6-carboxyfluorescein (FAM) reporter and a 3′ 6-carboxy-*N,N,N',N'*-tetramethylrhodamine (TAMRA) quencher. One primer/probe set targeted WNV E (forward primer 5′-TCAGCGATCTCTCCACCAAAG-3′, reverse primer 5′-GGGTCAGCACGTTTGTCATTG-3′, and probe 5′-FAM-TGCCCGACCATGGGAGAAGCTC-TAMRA-3′). Another primer/probe set targeted WNV NS5 (forward primer 5′-GCTCCGCTGTCCCTGTGA-3′, reverse primer 5′-CACTCTCCTCCTGCATGGATG-3′, and probe 5′-FAM-TGGGTCCCTACCGGAAGAACCACGT-TAMRA-3′). A sample was considered positive if the cycle threshold was ≥38. Some of the tissues were only positive for the WNV E gene; this was not unexpected since the assay for the E gene is more sensitive than the assay for the NS5 gene [45], [46]. RNA standards were *in vitro* transcribed from WNV cDNA and quantified as WNV genome copies, and 10-fold dilutions from 50 to 500,000 WNV genome copies were used as a standard curve. For RNA extracted from tissues, a third reaction was performed using a primer/probe set for a housekeeping gene (TaqMan® Rodent GAPDH, Applied Biosystems, Inc.). Rodent GAPDH standards (Applied Biosystems, Inc.) were used at 0.005 to 50 pg in 10-fold dilutions. Values for GAPDH and for WNV genome copies, using results for the primer-probe set targeting the E gene, were calculated by linear regression of the standard curves (Applied Biosystems, Inc.).

### Microsphere immunoassay for antibody to WNV

Sera collected from mice (Table S1) were analyzed for WNV-specific antibody by fluorescent MIA at a dilution of 1∶100 as previously described [34]. Briefly, microspheres (Luminex Corporation, Austin, TX) were coated with a recombinant WNV E antigen or a recombinant WNV NS5 antigen [34], [47]. Serum samples were diluted and incubated with the WNV E and WNV NS5 microspheres at 37°C for 30 minutes in darkness. After extensive washing, secondary polyvalent goat anti-mouse immunoglobulins (IgG, IgA, and IgM) conjugated to red-phycoerythrin were added to the samples, incubated for 30 minutes, and washed twice. The fluorescence intensity of the microspheres and MFI of the secondary antibody bound to the microspheres were analyzed with a Luminex 100 instrument (Luminex Corporation). Serum samples with MFI values greater than the cutoff were considered positive. Cutoff values were determined for each lot of beads and were based on the average MFI of serum samples from mock-inoculated mice plus three standard deviations (Microsoft®Office Excel, Microsoft Corporation, Seattle, WA). Cutoff values were: 1) MFIs of 20 and 860 for WNV E and WNV NS5 assays, respectively, for serum samples from 1, 2, 4, 6, 9 and 13 mo p.i.; 2) MFIs of 20 and 370 for WNV E and WNV NS5 assays, respectively, for serum samples from 16 mo p.i.. The data are shown as whisker-box plots with high and low values represented at the ends of the bars (GraphPad, San Diego, CA). The box represents the middle two quartiles (25th to 75th percentile) with the median represented as the horizontal line inside the box. A two-sided paired t test was used to analyze MFI data in the same mice at different time points (GraphPad).

### Virus neutralization assays

Sera collected from mice were also analyzed for neutralizing antibody using a PRNT. Due to limitations in sample size from mice that were bled from a peripheral vein, PRNTs were performed on sera from euthanized mice (1, 2, 4, 6, 9 and 16 mo p.i.) and from mice bled at 13 mo p.i. At 13 mo p.i., mice were bled two times one week apart, and the two serum samples were pooled together for each individual mouse, resulting in sufficient volume for the assay. All sera were heat-inactivated at 56°C for 1 hour prior to testing. Two-fold dilutions of the serum samples, starting at a final dilution of 1∶20, were incubated overnight at 4°C with 200 PFU of WNV. The virus-serum samples and appropriate controls were added to Vero cell monolayers in 6-well plates and incubated for 1 hour at 37°C, 5% CO_2_. Primary overlay (0.6% oxoid agar in supplemented medium) was added to each well, and plates were incubated for 2 days at 37°C, 5% CO_2_. On day 2, secondary overlay (0.6% oxoid agar in supplemented medium plus 0.33% Neutral Red) was added to each well. Plates were incubated overnight at 37°C, 5% CO_2_, and plaques were counted. End point titers were determined for the highest dilutions that inhibited 90% of viral plaques (PRNT_90_) and 50% of viral plaques (PRNT_50_). Titers of <1∶20 were considered negative. The data are shown as whisker-box plots with high and low values represented at the ends of the bars (GraphPad). The box represents the middle two quartiles (25th to 75th percentile) with the median represented as the horizontal line inside the box.

### Histopathology

Brains were harvested from mice that were sacrificed in the persistence study and processed for histology as follows. The brains were cut on the median plane, and one-half was placed in 10% formalin. After fixation for 1 week, tissues were paraffin embedded, and 6 µm sections were cut on a microtome (Leica Microsystems, Inc., Bannockburn, IL). Each section from a block contained representative areas of all brain regions. Slides were stained with hematoxylin and eosin. A board-certified veterinary pathologist (M.J.B) evaluated the brains (one slide per mouse) for the presence of histologic lesions, using mock-inoculated mice from each time point as comparisons. No histologic lesions were observed in mock-inoculated mice.

### Cyclophosphamide treatment

B6 mice were inoculated s.c. with diluent alone (n = 8) or with 10^3^ PFU of WNV (n = 22) and monitored as described above. The treatment regimen for cyclophosphamide was based on a previously published protocol [48]. On day 28 p.i., surviving mice were divided into two treatment groups. Group 1 was treated intraperitoneally with 5 mg cyclophosphamide (approximately 200 mg/kg, Sigma-Aldrich, St. Louis, MO) in endotoxin-free PBS and consisted of four mock-inoculated and ten WNV-inoculated mice. Group 2 was treated intraperitoneally with vehicle (endotoxin-free PBS) and consisted of four mock-inoculated and nine WNV-inoculated mice. A second treatment was administered on day 33 p.i.. Seventeen days after the first treatment, three WNV-inoculated mice from each treatment group and one mock-inoculated mouse from group 1 were sacrificed, and the following tissues were harvested: serum, skin–inoculation site (left rear footpad), lymph nodes (both popliteal and both inguinal lymph nodes pooled together), spleen, kidney, spinal cord, and brain. Tissues were processed for virus isolation and real-time RT-PCR assays as described above. A one-sided Fisher's exact test was used to compare the number of positive mice and the number of positive tissues in each treatment group (GraphPad). The remaining mice were monitored for clinical disease until the end of the study at 14 weeks p.i.

## Supporting Information

Figure S1 Experimental design. Flowchart depicting tissue processing for virus isolation and RNA extraction.
(0.23 MB TIF)

Table S1 Study design for WNV persistence in mice. The table provides the numbers of mice bled or sacrificed at each time point, including the number of mice with and without clinical disease during acute phase of disease (7 to 14 days p.i.).
(0.04 MB DOC)
