# Supplementary figures and images for: Persistence of West Nile Virus in the Central Nervous System and Periphery of Mice
Source: PLoS One. 2010 May 14;5(5):e10649. doi: 10.1371/journal.pone.0010649 (PMC2871051; doi:10.1371/journal.pone.0010649)

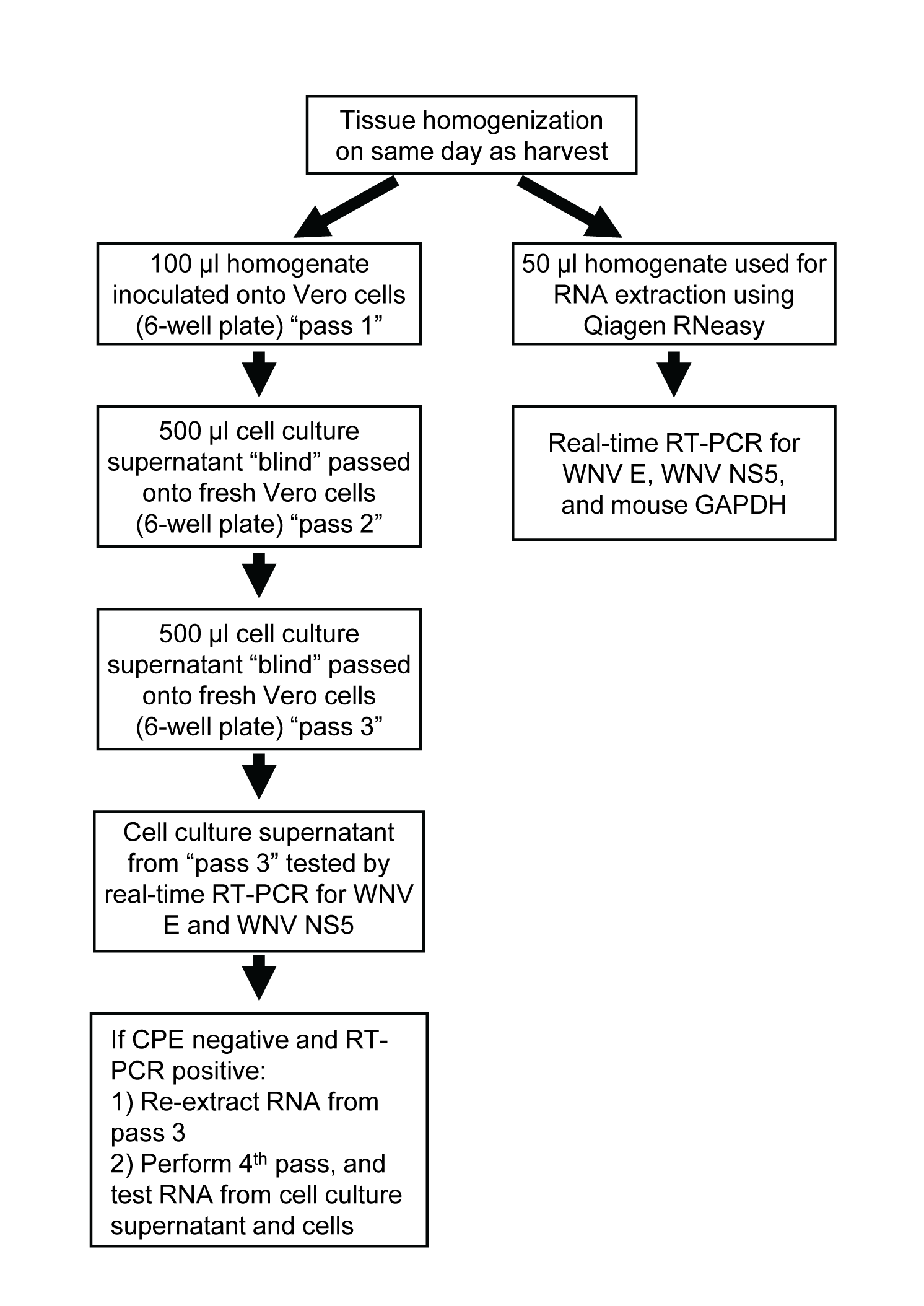

Supplement: Figure S1 — Experimental design. Flowchart depicting tissue processing for virus isolation and RNA extraction. (0.23 MB TIF) [file pone.0010649.s001.tif]
