# Supplementary material for: Persistence of West Nile Virus in the Central Nervous System and Periphery of Mice
Source: PLoS One. 2010 May 14;5(5):e10649. doi: 10.1371/journal.pone.0010649 (PMC2871051; doi:10.1371/journal.pone.0010649)
Supplement: Table S1 — Study design for WNV persistence in mice. The table provides the numbers of mice bled or sacrificed at each time point, including the number of mice with and without clinical disease during acute phase of disease (7 to 14 days p.i.). (0.04 MB DOC) [file pone.0010649.s002.doc]

**Table S1. Study design for WNV persistence in mice.**

| **Time p.i. (mo)** | **No. WNV-inoculated mice (no. with clinical disease^1^)** | **No. mock-inoculated mice** | **Procedure** |
| --- | --- | --- | --- |
| 1 | 64^2^ (6) | 8 | Bled for serologic assays |
| 1 | 8 (1) | 1 | Sacrificed for virus isolation, WNV RT-PCR, and histopathology |
| 2 | 40 (3) | 5 | Bled for serologic assays |
| 2 | 8 (1) | 1 | Sacrificed for virus isolation, WNV RT-PCR, and histopathology |
| 3 | 9 (1) | 1 | Bled for serologic assays |
| 3 | 9 (1) | 1 | Sacrificed for virus isolation and WNV RT-PCR |
| 4 | 39 (3) | 5 | Bled for serologic assays |
| 4 | 8 (0^3^) | 1 | Sacrificed for virus isolation, WNV RT-PCR, and histopathology |
| 6 | 31 (3) | 4 | Bled for serologic assays |
| 6 | 8 (1) | 1 | Sacrificed for virus isolation, WNV RT-PCR, and histopathology |
| 9 | 23 (2) | 3 | Bled for serologic assays |
| 9 | 8 (1) | 1 | Sacrificed for virus isolation, WNV RT-PCR, and histopathology |
| 13 | 15 (1) | 2 | Bled for serologic assays (serum pooled from two bleeds one week apart for each individual mouse) |
| 16 | 15 (1) | 2 | Bled for serologic assays |
| 16 | 15 (1) | 2 | Sacrificed for virus isolation and WNV RT-PCR |

^1^ Number of mice with clinical disease during acute phase of disease (7 to 14 days p.i.). ^2^ 82 mice were inoculated with WNV; there was 22% mortality, resulting in 64 surviving mice. ^3^ One mouse in this group showed repetitive motions and over grooming at the time of sacrifice.
